# A chromosome 1q22 microdeletion including *ASH1L* is associated with intellectual disability in a Chinese family

**DOI:** 10.1186/s13039-020-00483-5

**Published:** 2020-06-04

**Authors:** Hui Xi, Ying Peng, Wanqin Xie, Jialun Pang, Na Ma, Shuting Yang, Jinping Peng, Hua Wang

**Affiliations:** 1 NHC Key Laboratory of Birth Defect for Research and Prevention, Hunan Provincial Maternal and Child Health Care Hospital, Changsha, Hunan Province P. R. China; 2 The Prenatal Diagnosis Center of Hunan Province, Hunan Provincial Maternal and Child Health Care Hospital, Changsha, Hunan Province P. R. China; 3 Department of Medical Genetics, Maternal and Child Health Care Hospital of Shaoyang City, Shaoyang, Hunan Province P. R. China

**Keywords:** Intellectual disability, 1q22, *ASH1L*, Microdeletion, Prenatal diagnosis

## Abstract

**Background:**

Copy number variants (CNVs) associated with developmental delay and intellectual disability (DD/ID) continue to be identified in patients. This article reports identification of a chromosome 1q22 microdeletion as the genetic cause in a Chinese family affected by ID.

**Case presentation:**

The proband was a 19-year-old pregnant woman referred for genetic counseling and prenatal diagnosis at 18 weeks of gestation. She had severe ID with basically normal stature (height 154 cm [0 SD], weight 61 kg [− 0.2 SD], and head circumference 54 cm [− 1.12 SD]). Her distinctive facial features included a prominent forehead; flat face; flat nasal bridge and a short upturned nose; thin lips; and small ears. The proband’s father was reported to have low intelligence, whereas her mother was of normal intelligence but with scoliosis. Chromosome microarray analysis (CMA) reveals that the proband, her father and the fetus all carry a 1q22 microdeletion of 936.3 Kb (arr[GRCh37] 1q22 (155016052_155952375)×1), which was not observed in her mother and paternal grandparents and uncles, suggesting a de novo mutation in the proband’s father. The microdeletion involves 24 OMIM genes including *ASH1L* (also known as *KMT2H* and encoding a histone lysine methyltransferase). Of note, haploinsufficiency of *ASH1L* has been shown to be associated with neurodevelopmental disorders. Based on the inheritance of the detected CNV in the pedigree and similar CNVs associated with ID in public databases (Decipher, DGV and ClinVar) and literature, the detected CNV is considered as pathogenic. The family chose to terminate the pregnancy.

**Conclusions:**

The identified 1q22 microdeletion including *ASH1L* is pathogenic and associated with ID. This case broadens the spectrum of ID-related CNVs and may be useful as a reference for clinicians.

## Background

DD/ID refers to a large group of developmental disorders characterized by significant limitations in intellectual functioning and adaptive behaviors [1]. The onset of DD/ID usually is observed before the age of 18 years. Patients at the age of 5 years or younger who present with reasoning and learning difficulties and motor developmental delay are diagnosed as DD, whereas those who become symptomatic at the age over 5 years are regarded as ID [2]. DD/ID, with an estimated incidence of 1–3%, can be highly heterogeneous in clinical phenotype and genetic etiology [2]. About 25–50% DD/ID is associated with genetic alteration, such as 21, 18, or 13 trisomy or submicroscopic deletion/duplication [3].

CMA is featured by whole genome coverage, high resolution and rapid detection [4]. It has been recommended as the first-line clinical diagnostic test for individuals with unexplained DD/ID, autism spectrum disorders (ASDs) or multiple congenital anomalies (MCA) [5]. CMA can not only detect submicroscopic chromosomal imbalances, but also delineate the size and gene content of the detected segment. This is crucial for phenotype/genotype correlation and for identifying candidate genes involved in the development of certain anomalies [6].

The present case documented the clinical phenotype and genetic analysis of a Chinese family affected by ID using CMA.

## Case presentation

**Fig. 1:**
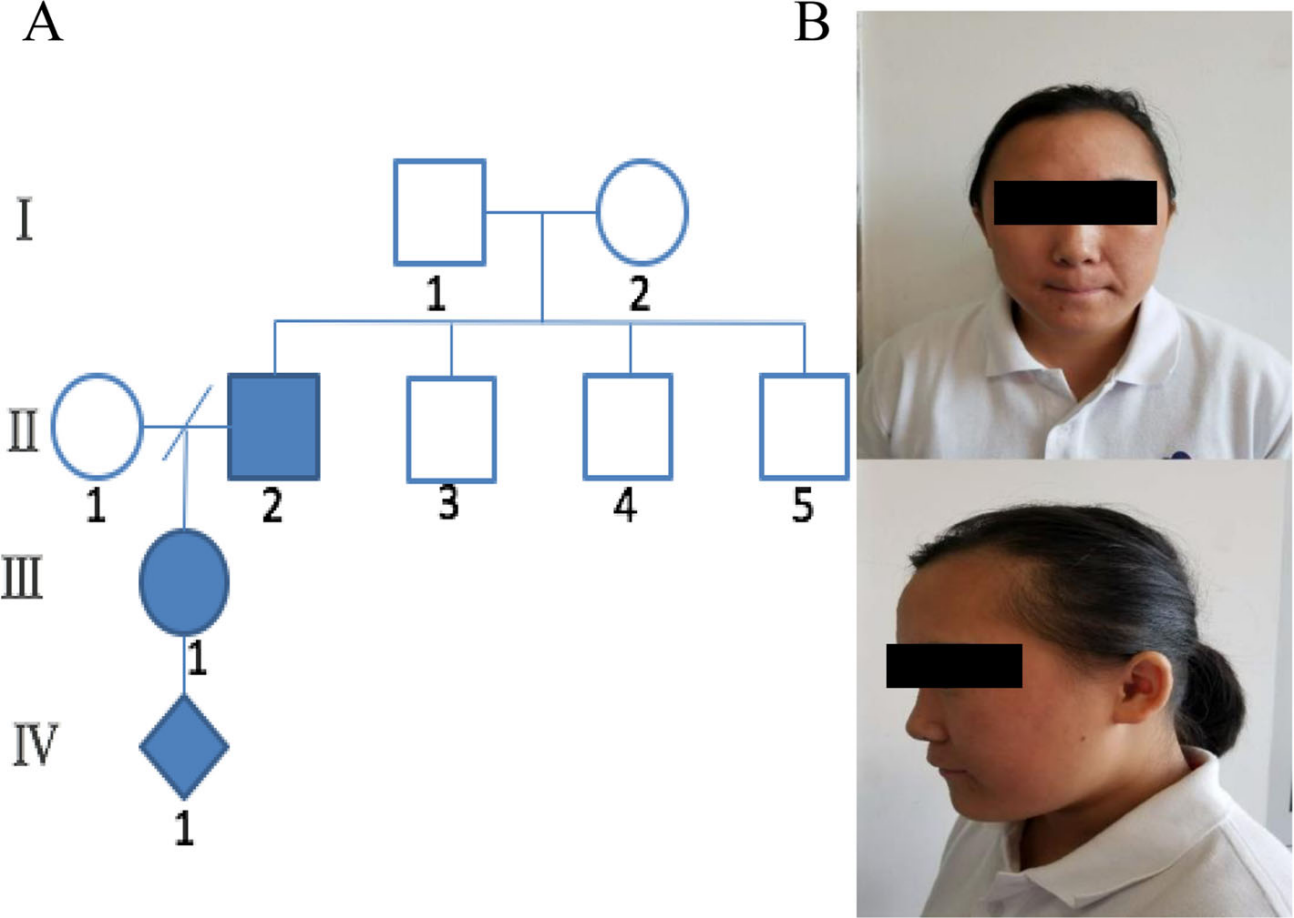
The pedigree (**a**) of a Chinese family affected by intellectual disability and facial features of the proband (**b**). The facial features of the proband (III-1) included a prominent forehead; flat face; flat nasal bridge and a short upturned nose; thin lips; and small ears

The proband was a 19-year-old pregnant woman who was referred to the department of medical genetics at the hospital for prenatal diagnosis due to a family history of intellectual disability. She was delivered vaginally at full-term. During the neonatal period, she was hypotonic and very passive. Her growth milestones were recalled. She walked at 1 year and 8 months of age, and learned to say “mama” at 2 years. She talked at nearly 3 years and showed severe ID. She began the first period of menstrual at the age of 13 years, and got married at 18 years. She was in pregnancy at 18 weeks’ gestation when referred for genetic counseling and prenatal diagnosis. She had facial dysmorphism including a prominent forehead; flat face; flat nasal bridge and a short upturned nose; thin lips; and small ears (Fig. 1). Examinations in psychological clinic showed that her height was 154 cm [0 SD]; weight was 61 kg [− 0.2 SD]; and head circumference was 54 cm [− 1.12 SD] [7, 8]. Her IQ score was 32 as accessed by the Wechsler Adult Intelligence Scale-Revised China (WAIS-RC). In terms of the scale, a score ≤ 75 is considered as low intelligence, and a score of 32 suggests severe ID. Reportedly, she was able to care for herself in daily life. Clinical observation showed that she was introverted; seldom talked; had dementia and social dysfunction without depression and anxiety. Both her electroencephalogram (EEG) and brain MRI result were normal. No history of heart diseases was noted. Her father was reported to have low intelligence, but an on-site examination for her father was not achieved. Her mother was of normal intelligence but had scoliosis. Her paternal grandparents and uncles and the fetus’ father had no noticeable congenital anomalies.

**Fig. 2:**
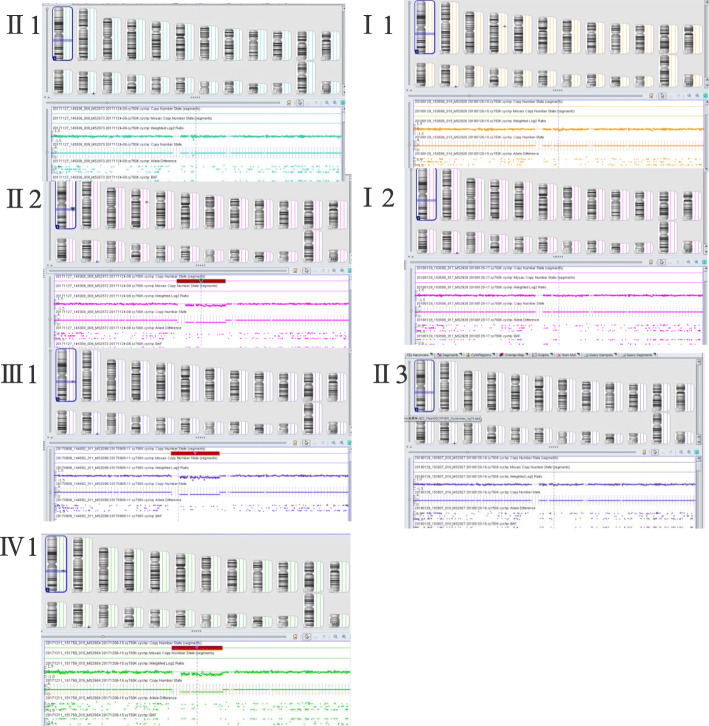
The SNP-array results of tested members from the Chinese family affected by intellectual disability. The proband (III-1), her father (II-2) and the fetus (IV-1) all contain a 936.3 kb heterozygous deletion of chromosome 1q22 (arr[GRCh37] 1q22 (155016052_155952375)×1). No significant CNVs were identified in the proband’s mother (II-1), grandparents (I-1 and -2) and paternal uncle II-3

Written informed consent was obtained from the family (The proband was under the guardianship of her mother). Peripheral blood for each participant and the amniotic fluid of the proband were drawn for genetic testing. Heart rate, blood pressure and electrocardiogram of the proband were monitored before amniocentesis. Conventional G-banded karyotype analysis showed a normal female karyotype (46,XX) in the proband. However, CMA using the CytoScan 750 K Array from Affymetrix (Thermo Fisher Scientific, USA) revealed a 936.3 kb heterozygous deletion of chromosome 1q22 (arr[GRCh37] 1q22 (155016052_155952375)×1) in the proband (Fig. 2). The microdeletion was also detected in the proband’s father (II-2) and the fetus (IV-1), but absent in her mother (II-1), grandparents (I-1, I-2) and paternal uncles (II-3, II-4). Other members within the pedigree were not tested. For all tested individuals, no other CNVs were detected except known polymorphisms (frequency > 1%).

In search of public databases (Decipher, DGV and ClinVar) and literature, a few cases reported copy number losses of the 1q22 region including *ASH1L* and the associated phenotypes including ID with MCA (Table 1). Furthermore, haploinsufficiency of *ASH1L* is strongly associated with DD/ID and MCA in multiple individual cases [9, 10]. On the basis of these observations, plus the co-segregation of genotype and phenotype in the current case, the detected CNV is considered to be pathogenic, being in line with the guidelines from the American College of Medical Genetics (ACMG) [11]. After full consideration, the family chose to terminate the pregnancy.

**Table 1:** 1q22 microdeletions/duplications including *ASH1L* from public databases and literature

Database/ Literature	Index ID	Variant	Size	Inheritance	Phenotype(s)	Clinic Significance
This report	/	[GRCh37] 1q22 (155016052 _155952375)×**1**	936.3 Kb	Paternal	Intellectual disability, behavioral problems, forehead, flat face, flat nasal bridge, short upturned nose, thin lips, small ears.	Pathogenic
Faundes et al.^*^	/	[GRCh37] 1q22 (155271366 _155804269)×**1**	532.9 Kb	De novo	Intellectual disability, behavioral problems, cryptorchidism and blocked nasolacrimal duct, microcephaly,	/
Decipher	249,031	[GRCh37] 1q22 (154292095 −155569326)×1	1.28 Mb	De novo	Delayed speech and language development, hypertelorism, intellectual disability, long face, low-set ears	Unknown
Decipher	255,240	[GRCh37] 1q22 (155192986 _156108069)×**1**	915.08 Kb	Unknown	Broad nasal tip, delayed speech and language development, dental malocclusion, intellectual disability, low-set ears, malar flattening, narrow palate, nasal speech, posteriorly rotated ears	Unknown
Decipher	359,103	[GRCh37] 1q22 (154687479 _156014014)×1	1.33 Mb	De novo	Abnormality of the nervous system, autism, scoliosis	Likely pathogenic
DGV	esv33869	[GRCh37] 1q22 (155223283 _155917961)×**1**	694.6Kb	/	/	/
DGV	dgv3n68	[GRCh37] 1q22 (155094978 _155313409)×1	218.4 Kb	/	/	/
ClinVar	VCV000659609.1	[GRCh37] 1q22 (155294636 _155452240)×**1**	157.6Kb	/	/	Pathogenic
Decipher	251,442	[GRCh37] 1q22 (155264908 _156495512)×**3**	1.23 Mb	Inherited	Cryptorchidism, delayed speech and language development, intellectual disability, strabismus	Unknown
ClinVar	VCV000253835.1	[GRCh37] 1q22 (155412745 _155755215)×**3**	342.47 kb	/	Attention deficit hyperactivity disorder	Uncertain

Selected deletion/duplication cases are listed. “/”, information not provided; “*”, reference [9]

## Discussion

The identified microdeletion in the proband involves 43 known genes, of which 24 were OMIM genes, including *ADAM15* (OMIM: 605548), *EFNA4* (OMIM: 601380), *EFNA3* (OMIM: 601381), *EFNA1* (OMIM: 191164), *SLC50A1* (OMIM: 613683), *DPM3* (OMIM: 605951), *TRIM46* (OMIM: 600986), *MUC1* (OMIM: 158340), *THBS3* (OMIM: 188062), *MTX1* (OMIM: 600605), *GBA* (OMIM: 606463), *SCAMP3*(OMIM: 606913), *CLK2* (OMIM: 602989), *HCN3*(OMIM: 609973), *PKLR* (OMIM: 609712), *FDPS* (OMIM: 134629), *ASH1L* (OMIM: 607999), *YY1AP1* (OMIM: 607860), *DAP3* (OMIM: 602074), *GON4L* (OMIM: 610393), *SYT11* (OMIM: 608741), *RIT1* (OMIM: 609591), *RXFP4* (OMIM: 609043), and *ARHGEF2* (OMIM: 607560). Among these OMIM genes, *TRIM46*, *CLK2*, *ASH1L*, *GON4L* and *ARHGEF2* are marked by a high probability of being loss of function intolerant (pLI ≥ 0.9), in contrast to other genes with a moderate (*RIT1* pLI: 0.67) or low probability. Therefore, haploinsufficiency of the genes with high pLI is particularly concerned. In OMIM database, the phenotypes of heterozygous loss of *TRIM46*, *CLK2*, *GON4L* and *ARHGEF2* in humans have not been fully documented, whereas a handful of individual patients featured by intellectual disability are reported to carry a heterozygous nonsense or frame-shift mutation of *ASH1L*, which results in truncated and nonfunctional protein [12–14]. The documented cases highly support that alteration in gene dosage of *ASH1L* is associated with neurodevelopmental disorders. In more recent studies, additional de novo loss of function variants of *ASH1L* have been identified, and all patients presented with mild to severe DD/ID [10]. Interestingly, a 532.9 kb heterozygous deletion (arr[GRCh37] 1q22 (155271366_155804269)×1) was found in a 7-year-old boy who had microcephaly and severe ID with MCA [9, 10]. Notably, the microdeletion identified in the present case fully encompasses the 532.9 kb segment and involves more OMIM genes. Based on these observations, the 1q22 microdeletion of 936.3 Kb including *ASH1L* is regarded as pathogenic and associated with ID. Importantly, not only micro-deletions, but also duplications within 1q22 may cause neurodevelopmental abnormalities (Table 1).

Though the pathogenicity of *ASH1L* haploinsufficiency is evident, contribution of other OMIM genes (e.g. *CLK2*, *ARHGEF2* and *RIT1*) to the phenotypes in this case cannot be fully excluded [15–17]. It is noteworthy that some clinical features of the proband resemble certain phenotypes such as broad forehead, broad nasal bridge, and learning/intellectual disabilities described in Noonan syndrome (NS), an autosomal-dominant disorder [15]. In a more recent report on the molecular and phenotypic spectrum of a Chinese NS cohort (*n* = 103), 6 out of 7 patients with *RIT1* mutation presented with various congenital heart defects, and a high rate (4 out of 7) of hypertrophic cardiomyopathy was observed in *RIT1* mutation-positive patients [18]. With regard to the proband in this report, although cardiac imaging examination was not performed, heart rate, blood pressure and electrocardiogram were recorded prior to ultrasound-guided amniocentesis. The normal readouts plus no previous history of heart disease suggested that it was unlikely that the patient had cardiomyopathy. Taking the potential involvement of multiple functional genes into account, it remains to be further elucidated whether this 1q22 microdeletion causes contiguous gene syndrome.

*ASH1L* encodes a histone lysine methyltransferase that catalyzes mono- and di-methylation of histone 3 lysine 36 (H3K36). This gene is highly expressed in both embryonic and adult human brains [19]. An inadequate amount of ASH1L protein due to copy number loss of the gene may affect epigenetic regulation of the expression program involved in embryo and brain development. Animal model studies show that mice in homozygosity of a hypomorphic *ASH1L* allele exhibited growth insufficiency, skeletal transformations and impaired fertility associated with developmental defects of reproductive organs [20]. In this case, the 1q22 microdeletion including *ASH1L* resulted from a de novo mutation in the proband‘s father. Phenotipically, both the proband and her father were fertile, suggesting that haploinsufficiency of *ASH1L* may not cause infertility in humans.

In summary, the present case shows the clinical phenotype of the proband in a Chinese family affected by ID and identification of a 1q22 microdeletion including *ASH1L* as the genetic cause in the pedigree. This case broadens the spectrum of ID-related CNVs and may be useful as a reference for clinicians. Apart from clinical findings, certain social and ethic issues also are brought into our sight by the case. For instance, was the pregnancy of the proband’s own free will? How should the fate of the fetus be decided? Though it may take time to find out the best solutions for the scenario resembling the case, there is no doubt that more social care is demanded with regard to women patients with intellectual disability.

## Data Availability

The datasets used and/or analyzed during the current study are available from the corresponding author on reasonable request.
